# Classroom-based cognitive behavioural therapy: a large-scale non-randomised controlled trial of the ‘Journey of the Brave’

**DOI:** 10.1186/s13034-021-00374-6

**Published:** 2021-04-24

**Authors:** Yuko Urao, Ikuyo Ohira, Takako Koshiba, Shin-ichi Ishikawa, Yasunori Sato, Eiji Shimizu

**Affiliations:** 1 Research Centre for Child Mental Development, Chiba University, 1-8-1 Inohana, Chuo-ku, Chiba, 260-8670 Japan; 2 Department of Psychology, Doshisha University, Kyoto, Japan; 3 Department of Preventive Medicine and Public Health, Keio University School of Medicine, Tokyo, Japan; 4 United Graduate School of Child Development, Osaka University, Kanazawa University, Hamamatsu University School of Medicine, Chiba University and University of Fukui, Osaka, Japan

**Keywords:** Cognitive behavioural therapy, Anxiety, Classroom-based, Prevention approach

## Abstract

**Background:**

In Japan, ‘Journey of the Brave’, a cognitive behavioural therapy (CBT)-based anxiety preventive education programme, was previously developed and its effectiveness examined in two small-scale controlled trials. These studies had some limitations, including a small number of participants and not having regular classroom teachers as programme facilitators. Therefore, we conducted a large-scale controlled trial, with teachers as programme implementers.

**Methods:**

Twenty-seven elementary schools participated: 1622 and 1123 children were allocated to the intervention and control groups, respectively. The intervention group received a programme comprising ten 45-min sessions, while the control group underwent the regular school curriculum. Anxiety symptoms among participants were assessed using the Spence Children’s Anxiety Scale (SCAS) at three stages (pre-intervention, post-intervention, and follow-up).

**Results:**

Following primary analysis, estimated mean changes in SCAS from baseline to follow-up were − 4.91 (95% CI − 5.91, − 3.90) in the intervention group and − 2.53 (95% CI − 3.52, − 1.54) in the control group; the group difference was 2.37 (95% CI 1.42, 3.33, *p* < 0.0001). Children in the intervention group showed significant reduction in their anxiety score versus children in the control group.

**Conclusions:**

The results showed a statistically significant anxiety score reduction in the intervention group, thus verifying the programme’s effectiveness.

*Trial registration* The University Hospital Medical Information Network (UMIN): UMIN000032517. Registered 10 May 2018—Retrospectively registered, https://upload.umin.ac.jp/cgi-open-bin/ctr_e/ctr_view.cgi?recptno=R000037083

## Background

Anxiety disorders are the most common mental health conditions affecting both children and adolescents [1–3]. Many children with anxiety disorders are reportedly left untreated [3] and this influences their academic performance, interpersonal relationships, family relationships, social adjustment, and quality of life [4–7]. All anxiety disorders can impact adult functioning [8–10] and involve a high risk of other mental diseases, such as depression [11–14]. Therefore, efforts to design and implement prevention or early intervention programmes aimed at childhood anxiety problems are essential, due to the extraordinarily high social cost of anxiety and depression [15, 16].

The number of children who refuse to attend school is consistently increasing in Japan (e.g., over 140,000 in 2017) and teachers have a difficult time dealing with this issue. Preceding overseas studies have reported a relationship between anxiety and higher levels of school absenteeism among children [17–19]. Additionally, in Japan, the results of an annual survey of elementary and junior-high schoolteachers, conducted by the Ministry of Education, Culture Sports Science and Technology (MEXT), showed that ‘anxiety tendency’ was the most prevalentamong the subcategory of ‘factors related to the particular child’ as a cause of school absenteeism [20]. Thus, there is an urgent need for effective countermeasures to prevent school absenteeism among children with underlying anxiety problems.

School-based preventative approaches that target mental disorders may be approximately divided into two categories: targeted and universal approaches. While targeted approaches are aimed at high-risk children, universal approaches are for all children regardless of their individual risk status [21]. Universal prevention programmes in schools have various merits: wide reach, no screening, no stigma, enhanced mental health and reduction in presenting symptoms [22]. Conducting programme sessions during school hours is a preferred method because all children can receive mental and physical health education in their natural environment, thus making the school an appropriate location for preventive education programmes [23].

Recently, several meta-analyses were conducted comparing targeted versus universal approaches; they concluded that there was no significant difference between the two approaches [24–26]. Thus, it is appropriate and important to implement a high-quality preventive education programme with universal approach for children during their school hours, with the aim of preventing mental disorders.

The effectiveness of programmes dealing specifically with anxiety or depression of children has been supported by several systematic reviews [24, 25, 27–32]. Preventive programmes that have proven to be effective are mostly CBT based [30]. Originally, clinical evidence suggested CBT was effective as a treatment for patients with either anxiety or depression [33], but recently CBT is being implemented more as a preventive measure. Several systematic reviews reported that among all of the CBT-based universal preventive programmes, the FRIENDS programme developed by Doctor Barrett from Australia, was more effective than most other programmes [24, 28, 34]. As the World Health Organization (WHO) has recommended the FRIENDS, many studies have been conducted to verify its efficacy. However, to date, the results of these studies have been inconsistent, with their effect sizes varying substantially [37].

In England, Stallard et al. [23], reported a cluster randomised control trial (c-RCT) that implemented the FRIENDS in 41 elementary schools. A significant reduction in RCADS (the Revised Child Anxiety and Depression Scale) scores was only evident in the health-led (led by health professionals) group, with no significant difference found between the school-led and usual school curriculum groups (health-led vs. school-led, *p* = 0.0004, health-led vs. usual school provision, *p* = 0.043).

In Japan, two small scale intervention studies analysed the effectiveness of the FRIENDS programme as facilitated by school psychologists [35, 36]. Matsumoto and Shimizu [35] conducted a non-randomised trial, but the intervention group did not display significant score reduction, and the effect size was reported to be small. Kato and Shimizu [36] also did not find any significant effectiveness. Given these results, we developed the ‘Journey of the Brave’ programme (JOB) with the motivation that it is necessary to develop a programme that is effective even when it is facilitated by elementary school teachers in Japan. JOB considers the specific cultural and social backgrounds of Japanese children and matches the education system of Japan [37].

Before this study, we conducted two small-scale controlled trials [37, 38]. The pilot study involved 9–12 year-old children attending elementary schools that held ten weeks of JOB sessions at a community centre during after school hours [37]. A medium effect size was observed in Spence Children’s Anxiety Score (SCAS) in the intervention group of 13 children (parental evaluation) at 3-month follow-up (SCAS-P: change from baseline 9.709, 95% CI 5.79, 14.23, *p* = 0.0001). Subsequently, we conducted a second study targeting fifth-grade (10–11 years old) schoolchildren with a larger sample size (intervention group *n* = 31, control group *n* = 41) using classroom-based interventions in an elementary school. This study also found significant anxiety score reductions in the intervention group, with the effects again being maintained at 3-month follow-up [38].

Although both preceding studies demonstrated the feasibility and effectiveness of the JOB, they had various limitations; such as limited sample sizes and the fact that they were both facilitated by a member of the research group, not school personnel. To strengthen the evidence for the programme’s efficacy and to implement it in more schools, it became necessary to run the programme in a greater number of schools while using schoolteachers as facilitators. Thus, the objective of this study was to verify the effectiveness of the JOB when it is implemented in classrooms of elementary schools in various areas of Japan and facilitated by teachers; accordingly, we conducted a large-scale controlled trial.

## Material and methods

### Study design and participants

A large-scale non-randomised controlled trial was conducted at several Japanese elementary schools, between September 2017 and March 2018. In conducting the trial, we decided to undertake a large-scale study with a planned sample size of 2000 (1000 intervention and 1000 control) to minimize the dispersion between the two groups. This study was approved by the ethics committee of Chiba University.

As per the eligibility criteria of the study participants, we selected (1) 10- to 12-year-old children in the 5th or 6th grade (2) whose parents consented to participation, and (3) who were able to attend over 80% of the ten programme sessions.

In recruiting participating schools, we contacted member universities of the Koroma Minna Project (http://www.kodomo-minna.jp/) through their cooperating education boards. This project began as an initiative of the Ministry of Education, Culture, Sports, Science and Technology (MEXT), who delegated the authority to run it to ten universities to solve difficult mental health problems in the Japanese school system, such as school absenteeism and bullying. Each participating education board or university distributed an announcement letter soliciting participation, along with an application form and pamphlet explaining the content to jurisdictional elementary schools. Thirty schools willingly participated in this study. They were geographically separated into three different areas: Chiba, Tottori, and Kyoto prefectures.

Fifth (10- to 11-year-old) or sixth grade (11- to 12-year-old) children were assigned to either the intervention group, which received both programme sessions and the survey, or the control group which received normal classes and the survey. The schools managed the grouping with some variations: some schools that chose fifth graders as the intervention group had difficulty in assigning 6th graders as the control group because these students were exceptionally busy; as such, these schools were permitted to assign fourth graders as the control group.

The teachers selected as facilitators attended a 6-h facilitator workshop beforehand, prepared by the programme developers.

### Interventions

The programme consisted of ten standardised CBT sessions, each of 45 min. During the sessions each child took note of the learned content and wrote it in a workbook. The teachers conducted the sessions using workbooks and detailed facilitator manuals. A homework sheet was provided to help the children internalise the CBT-based knowledge and skills aimed at dealing with their anxiety problems by applying the classroom teachings and completing the assigned homework (Table 1).

**Table 1:** Session content of the JOB programme

Session	Content	Component of CBT
1	Understanding the four basic feelings	Psychoeducation (e.g., the role of negative feelings) Log positive feelings (mastery and pleasure technique)
2	Monitoring feelings of anxiety and setting goals	Psychoeducation (e.g., the role of anxious feelings) Monitor and log anxious feelings
3	Body reactions and relaxation	Psychoeducation (e.g., fight or flight response) Relaxation training (muscle and breathing relaxation)
4	Anxiety-level stages and stair step exposure	Psychoeducation (e.g., principle of gradual exposure) Develop stair step and face gradual exposure
5	Anxiety cognition model	Psychoeducation (e.g., cognitive model) Develop cognitive model
6	Identifying cognitive distortions and coping with rumination	Psychoeducation (e.g., dysfunctional cognitions) Develop meta-cognitive awareness and monitor maladaptive thoughts
7	Cognitive restructuring when anxious	Psychoeducation (e.g., cognitive restructuring) Implement cognitive restructuring
8	Assertiveness skills to reduce social anxiety	Psychoeducation (e.g., assertiveness) Develop assertiveness using DESC response
9 and 10	Summary	Review of all CBT techniques

The facilitators’ workshop included a mini lecture on CBT and programme content explanation in the first half, with facilitation role-plays and feedback in the second. The training seminars were held eight times between July and August 2017 and were mandatory for each facilitator. Each of the seminars was attended by 30–40 teachers.

The programme sessions were conducted as a part of the regular school curriculum, such as homeroom activity, general learning or health promotion, and physical exercise classes. Simultaneously, the control group continued with their regular school curriculum. Each school was responsible for programme completion within the academic year.

### Measurements

The primary outcome of this study was changes in the students’ SCAS Japanese version scores [39]. The SCAS [40] is an anxiety scale that has been shown to have good psychometric properties and has been commonly used with children of various cultural backgrounds in multiple countries. It contains six subscales: generalised anxiety disorder, separation anxiety disorder, social phobia, panic disorder and agoraphobia, obsessive–compulsive disorder, and fear of physical injury. Each item is rated on a 4-point scale in terms of its frequency, from ‘*never’* (0) to ‘*always’* (3). A total anxiety score is calculated by adding the 38 item’ scores, with a maximum possible score of 114. According to the developer of the SCAS (https://www.scaswebsite.com/index.php?p=1_9), a T score above 60 (SCAS above 40 for 8–11 year-old male children, above 50 for female children) is classified into an elevated anxiety group. According to Muris et al. [41], the mean SCAS score of children aged 7 to 12 years is 20.51(SD = 14.20); the 10% cut-off score is 42. According to Ishikawa, Sato, and Sasagawa [40], the average SCAS score of a sample of Japanese children (*n* = 1045, mean age = 12.01 years, SD = 1.81) was reported as 23.50 (boys: 19.06, girls: 27.89). Additionally, Ishikawa et al. [39] confirmed the reliability (Cronbach’sα = 0.94, test–retest reliability = 0.76, *p* < 0.001) and validity (correlation with Depression Self-Rating Scale: r = 0.47, *p* < 0.001) of the SCAS Japanese version.

The SCAS surveys were administered by the teachers during class time in the classrooms in three stages: pre-intervention (time 1), post-intervention (time 2), and 1- to 3-months post-intervention follow-up (time 3). Each survey was read by one of the classroom teachers to the children who completed the form simultaneously. Originally, the follow-up was set at 3 months post-intervention, but due to limitations of some schools, follow-up between 1 and 3- months was accepted. The control group completed the SCAS survey at the same time as the intervention group, enabling valid score comparisons.

### Statistical analysis

The facilitators converted the SCAS score data into a specialised format to ensure anonymity of the participants and sent the data to the research centre where it was analysed by the research specialists. Primary analysis was performed using a mixed-effects model for repeated measures (MMRM) with the intervention group, time, and interactions between intervention group and time as the fixed effects; an unstructured covariance matrix was used to model the within-subject error and the Kenward–Roger approximation was used to estimate the degrees of freedom. The MMRM analysis assumes that any missing data occur randomly. All comparisons in the model were planned, with all *p*-values two-sided. A *p*-value < 0.05 was considered statistically significant. All statistical analyses were performed using the SAS software program, version 9.4 (SAS Institute, Cary, NC, USA) and SPSS Version 22.0 (IBM, Armonk, New York, USA).

Data were examined to identify the number of participants whose scores were in the clinical range of anxiety. Based on their pre-intervention scores, participants were divided into two groups. Children with pre-intervention SCAS scores of 45 or above (which included the top 10%) were categorised as the high-anxiety group, while those with scores below 45 formed the regular-anxiety group; a sub-analysis was conducted to compare these two groups. This study was registered with the University Hospital Medical Information Network: UMIN000032517 (https://upload.umin.ac.jp/cgi-open-bin/ctr_e/ctr_view.cgi?recptno=R000037083).

## Results

The trial conducted between April 2017 and March 2018 enrolled 27 schools. In total, 1,583 children from the intervention group (male: *n* = 800, female: *n* = 821, missing *n* = 1) and 1,095 from the control group (male: *n* = 522, female: *n* = 569, missing *n* = 32) were the final participants of this study after removing the opt-outs. Although there was a statistically significant difference in the number of children between the two groups (χ^2^ = 90.71, df = 1, *p* < 0.001), there was no significant difference in the sex ratio between groups (χ^2^ = 0.527, df = 1, *p* = 0.468).

**Fig. 1:**
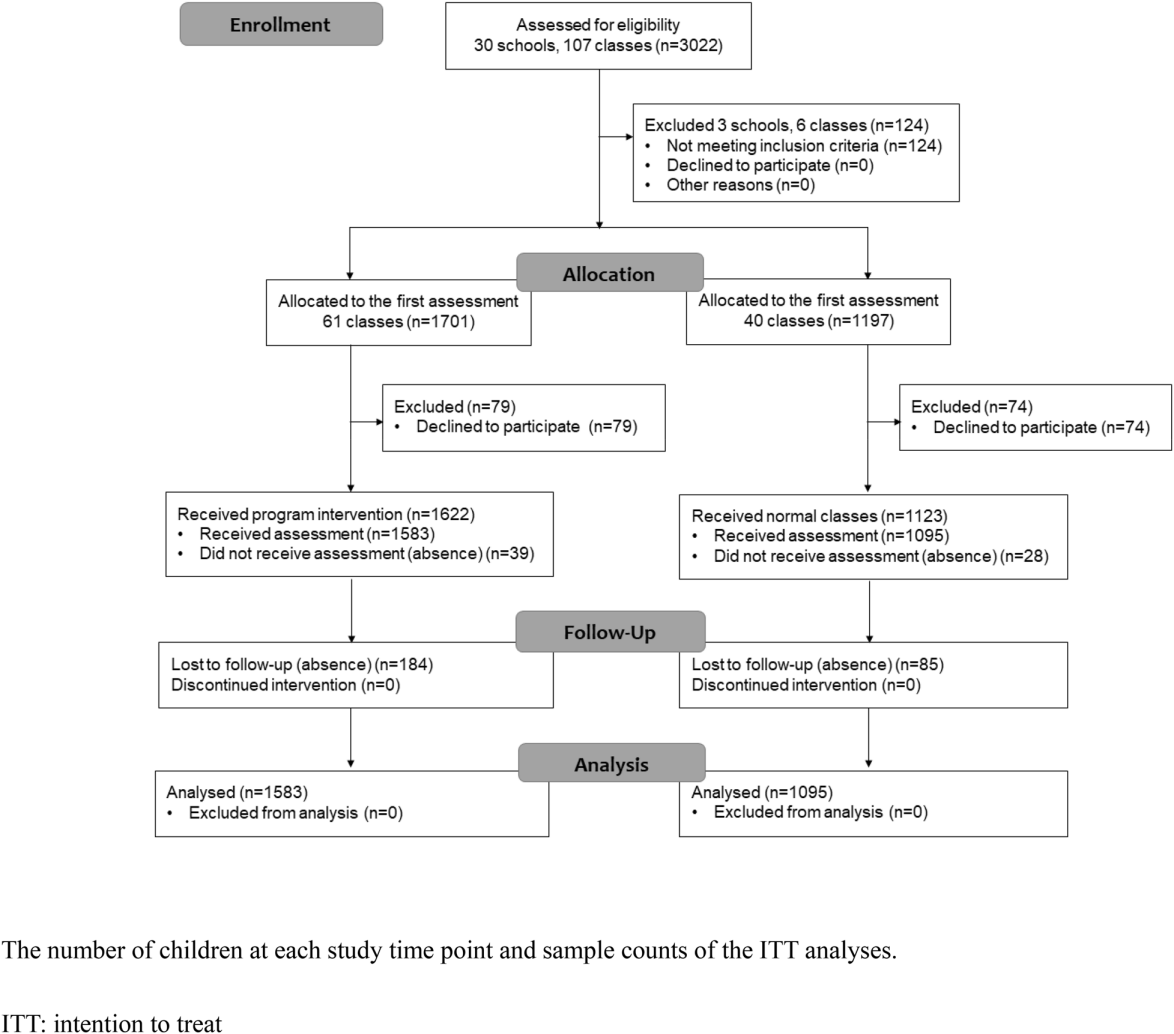
Trial profile. The number of children at each study time point and sample counts of the ITT analyses. *ITT* intention to treat

Although the frequency of the sessions varied among schools, the programme sessions were held once a week on average, between September 2017 and February 2018. There were winter holidays during the intervention, but they did not affect negatively to the continuality of the intervention. Figure 1 shows the number of children at each time point, as well as the sample count of the intention-to-treat analysis. Of the 2,745 children who participated, 2,678 completed the baseline assessment.

Table 2 shows the baseline SCAS score average by group, sex, region, and grade. There were no statistically significant differences between groups (*p* = 0.55) or geographical areas (*p* = 0.14) in baseline SCAS scores, but there were statistically significant differences by sex (*p* < 0.001). The SCAS score average of all children was 21.82 (*SD* = 16.54).

**Table 2:** Baseline SCAS score average by group, sex, region, and grade

	n	p-value	SCAS score (SD)	p-value	N	SCAS score (SD)	p-value
Sex							
Male	1320	0.44	19.17 (15.38)	.001	IG 783 CG 537	IG 19.68 (15.35) CG 18.41 (15.41)	.14
Female	1358		24.39 (17.21)		IG 800 CG 558	IG 23.59 (17.02) CG 25.25 (17.45)	.40
Region							
Chiba (East)	2115	< 0.01	21.49 (16.41)	.14	IG 1156 CG 957	IG 21.21 (16.11) CG 21.83 (16.78)	.38
Tottori (West)	424		23.09 (16.65)		IG 366 CG 58	IG 23.36 (16.89) CG 21.38 (15.08)	.36
Kyoto (Central)	139		22.94 (17.92)		IG 61 CG 78	IG 19.97 (16.37) CG 25.27 (18.83)	.08
Grade							
4th (9–10 y)	108	< 0.01	20.18 (17.92)	.02	IG- CG 108	IG- CG 20.18 (17.92)	–
5th (10–11 y)	1352		22.69 (16.85)		IG 595 CG 757	IG 23.03 (17.26) CG 22.43 (16.53)	.51
6th (11–12 y)	1218		20.99 (16.02)		IG 988 CG 228	IG 20.83 (15.69) CG 21.71 (17.40)	.48
Total	2678	–	21.82 (16.54)	–	IG 1583 CG 1095	IG 21.66 (16.32) CG 22.05 (16.85)	.54

*SCAS* Spence Children’s Anxiety Scale, *SD* standard deviation

**Fig. 2:**
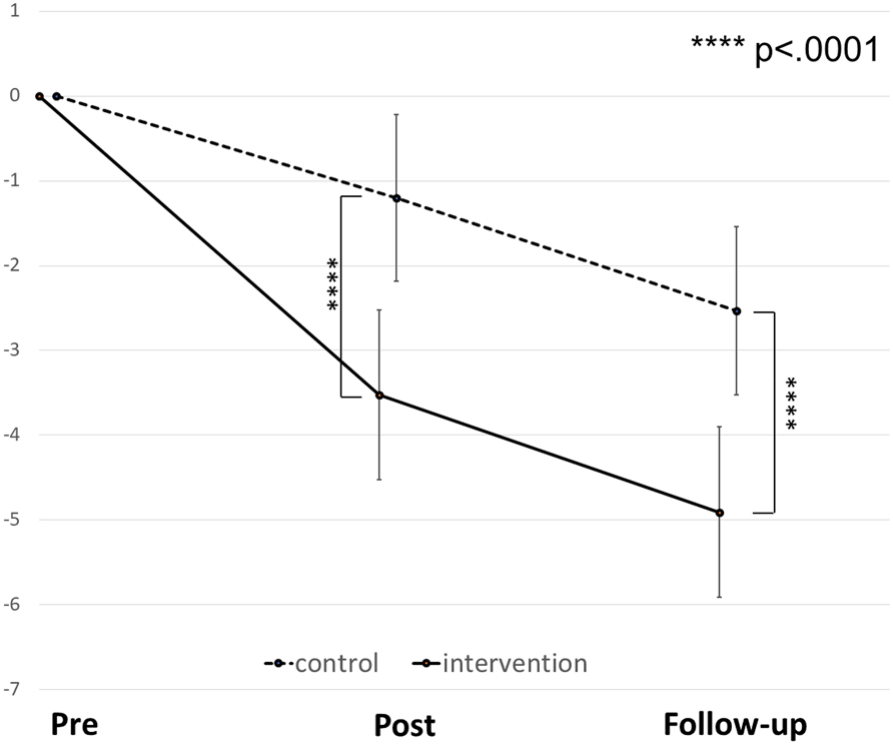
Changes in mean SCAS scores in intervention and control groups at each time point

Table 3 shows the means and standard deviations of the intervention and control groups’ SCAS scores at each time point. Figure 2 presents the results of the MMRM analysis of the SCAS scores. In the primary analysis of the SCAS scores, the adjusted mean changes from baseline to follow-up were − 4.91 (95% CI − 5.91, − 3.90) and − 2.53 (95% CI − 3.52, − 1.54) for the intervention and control groups, respectively; the difference between these groups was 2.37 (95% CI 1.42, 3.33, *p* < 0.0001).

**Table 3:** Means and standard deviations of SCAS scores at each time point

Group	Time 1 (pre)	Time 2 (post)	Time 3 (follow-up)
Intervention			
SCAS (*SD*)	21.66 (16.32)	18.87 (15.81)	17.37 (15.20)
*n*	1583	1490	1399
Control			
SCAS (*SD*)	22.05 (16.85)	21.22 (17.85)	19.79 (17.78)
*n*	1095	1050	1010

**Fig. 3:**
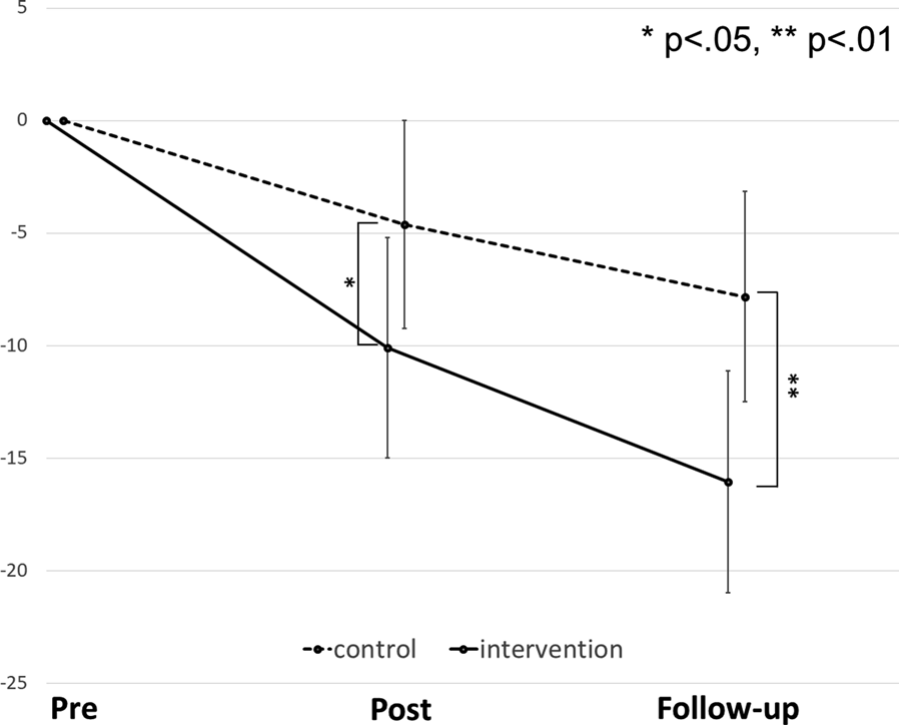
Changes in mean SCAS scores of high anxiety group children (SCAS ≧ 45 at baseline) of the intervention group and the control group at each time point

Table 4 shows the means and standard deviations of SCAS scores for children with high anxiety (SCAS ≥ 45 at baseline) at each time point. Figure 3 presents the results of the MMRM analysis of the high-anxiety intervention (*n* = 214) and control groups’ (*n* = 181) SCAS scores. In the secondary analysis of the SCAS scores, the adjusted mean changes were − 16.05 (95% CI − 20.99, − 11.12) and − 7.82 (95% CI − 12.51, − 3.14) for the intervention and control groups, respectively; the group difference was 8.23 (95% CI 3.63, 12.83, *p* = 0.001) (Table 5).

**Table 4:** Means and standard deviations of SCAS scores for children with high anxiety (SCAS ≥ 45 at baseline) at each time point

Group	Time 1 (pre)	Time 2 (post)	Time 3 (follow-up)
Intervention			
SCAS (*SD*)	52.33 (13.43)	42.51 (16.97)	37.42 (17.78)
*n*	214	198	185
Control			
SCAS (*SD*)	51.69 (11.32)	48.51 (18.57)	44.96 (21.96)
n	181	168	162

**Table 5:** Number and proportion of children with high anxiety (SCAS≧45) among all children

	Time 1 High/total (%)	Time 2 High/total (%)	Time 3 High/total (%)
Intervention	146/1579 (9.25%)	114/1473 (7.74%)	76/1387 (5.58%)
Control	124/1093 (11.34%)	114/1031 (11.06%)	106/992 (10.69%)

**Fig. 4:**
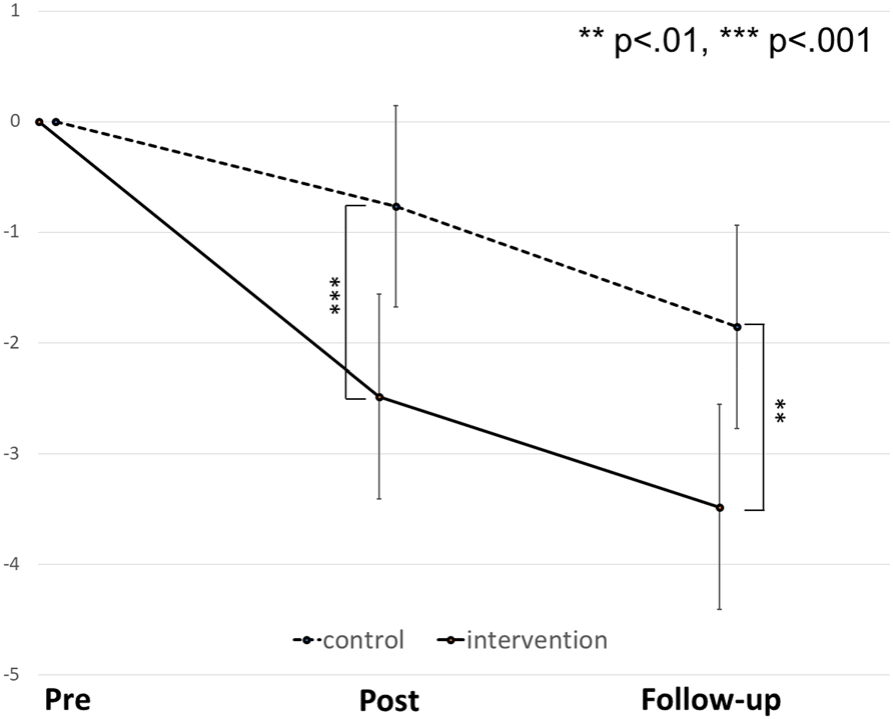
Changes in mean SCAS scores of low-anxiety group children (SCAS ≦ 44 at baseline) of the intervention group and the control group at each time point

Figure 4 presents the results of the MMRM analysis of the lower-anxiety (SCAS < 45 at the baseline) intervention (*n* = 1369) and control groups’ (n = 914) SCAS scores at each time point. In the secondary analysis of the SCAS scores, the adjusted mean changes were − 3.48 (95% CI − 4.41, − 2.56) and − 1.86 (95% CI − 3.41, − 1.56) for the intervention and control groups, respectively; the group difference was 1.63 (95% CI 0.74, 2.52, *p* = 0.0004).

## Discussion

This study is the largest-scale trial of a CBT-based anxiety prevention programme in Japan. The results demonstrated that ten 45-min JOB sessions conducted in Japanese elementary schools, delivered by specially trained teachers, were associated with a statistically significant anxiety score reduction among the participating students, when compared with a control group.

The first issue to consider is the baseline scores. In this study, the average SCAS baseline score of 2678 children is 21.82, which is lower than the 23.50 reported by Ishikawa et al. [39]. Although the reason is not clear, this may have been the result of the difference in survey timing, sample size, and most likely, geographical area. In fact, there were differences between scores of the three regions sampled in our study (Table 2). The average score of children from the Tottori area in the western part of Japan was 23.09, close to the average score reported by Ishikawa et al.; and school absenteeism in Tottori is among the highest in Japan. In contrast, children from Chiba (in eastern Japan) and Kyoto (in central Japan) were less anxious as compared with the country average. Thus, it is necessary to consider geographical differences in baseline scores. In future, it will be important to assess differences in intervention effectiveness according to the magnitude of baseline scores.

In our study, 10% of children were categorised into the high-anxiety group with baseline SCAS scores of above 45. In Japan, some children diagnosed with autism spectrum disorder (ASD) and/or attention deficit hyperactivity disorder (ADHD) attend regular school classes. Although the number of children with ASD or ADHD in this study is unknown, 6.5% of children in regular Japanese school classes fell into these categories according to a 2012 government estimate [42]. It has been reported that 40% of children with ASD experience complications of comorbid anxiety disorders [43]. This is in addition to the fact that children with high anxiety are more likely to develop more serious anxiety disorders, thus resulting in increased concern for their healthy development. When an anxiety prevention programme is conducted in schools using a universal approach, it is expected to curbing potential complications among children with developmental problems.

The next point that requires consideration is the interpretation of the SCAS score reduction after the program. The difference in score reduction between the intervention and control groups, although small (2.37 units), was meaningful. Most participants in universal preventive programs are children without any clinical problems such as anxiety disorder; that is, most children have a low SCAS score and are unlikely to experience a major score reduction. Nevertheless, that overall class and school average anxiety symptoms measured by SCAS significantly reduced indicates that the program was valuable for promoting children’s mental health.

Our sub-analysis indicated that although the score of the group of children with high anxiety significantly reduced after the intervention, the score of the control group was also below the cut-off (SCAS ≧ 45) at follow-up. When the percentage of children with high anxiety was calculated, although there was little change in the control group, a small change was present in the intervention group. This result indicates that the program may have helped to reduce the anxiety of some children in the high anxiety group. In contrast, some children with high anxiety did not demonstrate any score reduction. In this respect, Werner-Seidler et al. [25] noted, ‘how future studies may be improved is to take a stepped care approach where a universal programme is delivered in the first instance, and is followed up with a targeted programme for at-risk or symptomatic individuals who do not respond to the universal programme’. Consequently, researchers should be aware of the limitations of the universal approach; a perspective that pays more attention to children with urgent needs and takes a targeted approach is also needed.

The next point of interest is the program facilitator. In the preceding JOB studies, program sessions were facilitated by healthcare specialists [37, 38]; the current study was the first JOB verification study facilitated by classroom teachers. The systematic review by Fisak et al. [24] describes a significant difference in the results depending on who delivers the programme; a programme tends to be less effective if led by lay providers instead of being health-led. Thus, the significant effectiveness of the current teacher-led program is promising in its implications for the future promotions of this programme. One systematic review, conducted by Werner-Seidler et al. [25], reported no effect size differences when comparing who led the anxiety prevention programme and suggested that this may be because children feel more comfortable in the presence of their teachers rather than an outside professional whom they see as a stranger. Collins et al. [44] also emphasised the merit of teacher-led approaches as it is easier for teachers to notice the daily stress factors of children; if such factors become problematic, the teachers can easily apply their programme. This may be a reason for the significant score reductions among the children in the intervention group. In future, it will be necessary to conduct replication studies to confirm the effectiveness of JOB when the program is delivered by regular class teachers.

### Limitations

This study has some methodological limitations. The first issues pertain to the research design and sampling. Ideally, this type of research should be a RCT, but this was a non-randomised controlled trial. Regarding sampling, some schools had to assign 4th graders, whose characteristics may be different from those of 5th or 6th grade children as the control group to match the number of children in the two groups to the extent possible. This approach was not ideal.

This was the first large-scale control trial of the JOB and many schools chose to participate because they wished to cooperate. However, because of the participating teachers’ passion, they wished to try this with their students immediately for its potentially positive results. If this study used a c-RCT design, assigning half of the schools to the control group, there would have been less willingness of schools to participate. In future, if we wish to conduct a c-RCT in Japan, it would be necessary to not only initiate this programme throughout Japan, but also to secure organised cooperation from local education boards and the Japanese government (MEXT).

Second, although there was a guideline of session frequency of once a week, some schools adopted their own interval and frequency because of scheduling reasons. This may have biased the results to some extent. It is extremely difficult to secure class time for subjects that are not authorized in the study guidelines for Japanese schools because the curriculum detail is strictly set in the guidelines provided by MEXT. To ensure the rigour of future research, it would be necessary to secure the cooperation of MEXT and local education boards and set program session frequency so that the verification of effectiveness would be more reliable.

Third, the methods used to evaluate the programme implementation and effectiveness were insufficient; anxiety changes of the participants were measured using self-reported surveys and not evaluated using rigorous and objective tools, such as a diagnostic interview, and programme fidelity was not measured. In fact, there was some dispersion in the score changes by school. In future, rigorous adherence could be ensured by video recording actual sessions to ensure program fidelity. It will also be necessary to research the number of children with school absenteeism and/or with anxiety disorder when evaluating the effectiveness of preventive programmes.

Forth, the follow-up period was up to three months; long-term effects were not fully verified. Stallard et al. [22] stated that when evaluating a prevention programme, it is necessary to conduct a long-term follow-up assessment to determine whether the programme was effective. In Japanese elementary schools, a class member shuffle and class teacher change occurs annually. There is no system of collecting individual and yearly long-term follow-up data. Thus, it would be preferable to have the understanding and support of the government (MEXT) as well as local education boards with respect to the importance of conducting a long-term study, as well as the involvement of researchers who adopt a more long-term perspective.

## Conclusions

This was the first large-scale controlled trial of the effectiveness of the CBT-based anxiety prevention programme ‘Journey of the Brave’ as facilitated by classroom teachers in Japan. The trial was conducted in 30 Japanese elementary schools in different areas. A significant anxiety score reduction was confirmed for children in the intervention group compared with a control group. A future vision is to conduct a large-scale follow-up study and/or a c-RCT to judge programme effectiveness more rigorously.

## Data Availability

The datasets used and/or analysed during the current study are available from the corresponding author on reasonable request.

## Abbreviations

CBT: Cognitive behavioural therapy
SCAS: Spence children’s anxiety scale
MMRM: Mixed-effects model for repeated measures
ITT: Intention to treat
RCT: Randomized controlled trial
c-RCT: Cluster randomised control trial

## References

[1] Cartwright-Hatton S, McNicol K, Doubleday E (2006). Anxiety in a neglected population: prevalence of anxiety disorders in pre-adolescent children. Clin Psychol Rev.

[2] Merikangas KR, Nakamura EF, Kessler RC (2009). Epidemiology of mental disorders in children and adolescents. Dialogues Clin Neurosci.

[3] Merikangas KR, He JP, Burstein M, Swanson SA, Avenevoli S, Cui L (2010). Lifetime prevalence of mental disorders in US adolescents: results from the National Comorbidity Survey Replication-Adolescent Supplement (NCS-A). J Am Acad Child Adolesc Psychiatry.

[4] Langley AK, Bergman RL, McCracken J, Piacentini JC (2004). Impairment in childhood anxiety disorders: preliminary examination of the child anxiety impact scale–parent version. J Child Adolesc Psychopharmacol.

[5] Mogotsi M, Kaminer D, Stein DJ (2000). Quality of life in the anxiety disorders. Harv Rev Psychiatry.

[6] Nail JE, Christofferson J, Ginsburg GS, Drake K, Kendall PC, McCracken JT (2015). Academic impairment and impact of treatments among youth with anxiety disorders. Child Youth Care Forum.

[7] Van Ameringen M, Mancini C, Farvolden P (2003). The impact of anxiety disorders on educational achievement. J Anxiety Disord.

[8] Ollendick TH, King NJ (1994). Fears and their level of interference in adolescents. Behav Res Ther.

[9] Öst LG, Treffers PD (2001). Onset, course, and outcome for anxiety. Anxiety disorders in children and adolescents: research, assessment and intervention.

[10] Pine DS, Cohen P, Gurley D, Brook J, Ma Y (1998). The risk for early-adulthood anxiety and depressive disorders in adolescents with anxiety and depressive disorders. Arch Gen Psychiatry.

[11] Bienvenu OJ, Ginsburg GS (2007). Prevention of anxiety disorders. Int Rev Psychiatry.

[12] Copeland WE, Shanahan L, Costello EJ, Angold A (2009). Childhood and adolescent psychiatric disorders as predictors of young adult disorders. Arch Gen Psychiatry.

[13] Flannery-Schroeder EC (2006). Reducing anxiety to prevent depression. Am J Prev Med.

[14] Rice F, van den Bree MB, Thapar A (2004). A population-based study of anxiety as a precursor for depression in childhood and adolescence. BMC Psychiatry.

[15] Bodden DH, Dirksen CD, Bögels SM, Nauta MH, De Haan E, Ringrose J (2008). Costs and cost-effectiveness of family CBT versus individual CBT in clinically anxious children. Clin Child Psychol Psychiatry.

[16] DeKlyen M, Greenberg MT, Cassidy PR, Shaver (2008). Attachment and psychopathology in childhood. Handbook of attachment: theory, research, and clinical applications.

[17] Egger HL, Costello JE, Angold A (2003). School refusal and psychiatric disorders: a community study. J Am Acad Child Adolesc Psychiatry.

[18] Gonzálvez C, Kearney CA, Jiménez-Ayala CE, Sanmartín R, Vicent M, Inglés CJ, García-Fernández JM (2018). Functional profiles of school refusal behavior and their relationship with depression, anxiety, and stress. Psychiatry Res.

[19] Ingul JM, Nordahl HM (2013). Anxiety as a risk factor for school absenteeism: what differentiates anxious school attenders from non-attenders?. Ann Gen psychiatry.

[20] Ministry of Education, Culture, Sports, Science and Technology. Research result on various tasks of student guidance of pupils with problematic behaviour and school absenteeism in 2018; 2019. https://www.mext.go.jp/b_menu/houdou/31/10/1422020.htm. Accessed 15 Aug 2019.

[21] Haggerty RJ, Mrazek PJ (1994). Reducing risks for mental disorders: Frontiers for preventive intervention research.

[22] Stallard P, Skryabina E, Taylor G, Phillips R, Daniels H, Anderson R, Simpson N (2014). Classroom-based cognitive behaviour therapy (FRIENDS): a cluster randomised controlled trial to Prevent Anxiety in Children through Education in Schools (PACES). Lancet Psychiatry.

[23] Stallard P, Sayal K, Phillips R, Taylor JA, Spears M, Anderson R (2012). Classroom based cognitive behavioural therapy in reducing symptoms of depression in high risk adolescents: pragmatic cluster randomised controlled trial. BMJ.

[24] Fisak BJ, Richard D, Mann A (2011). The prevention of child and adolescent anxiety: a meta-analytic review. Prev Sci.

[25] Werner-Seidler A, Perry Y, Calear AL, Newby JM, Christensen H (2017). School-based depression and anxiety prevention programs for young people: a systematic review and meta-analysis. Clin Psychol Rev.

[26] Zalta AK (2011). A meta-analysis of anxiety symptom prevention with cognitive-behavioral interventions. J Anxiety Disord.

[27] Corrieri S, Heider D, Conrad I, Blume A, König HH, Riedel-Heller SG (2014). School-based prevention programs for depression and anxiety in adolescence: a systematic review. Health Promot Int.

[28] Johnstone KM, Kemps E, Chen J (2018). A meta-analysis of universal school-based prevention programs for anxiety and depression in children. Clin Child Family Psychol Rev.

[29] Moreno-Peral P, Conejo-Cerón S, Rubio-Valera M, Fernández A, Navas-Campaña D, Rodríguez-Morejón A (2017). Effectiveness of psychological and/or educational interventions in the prevention of anxiety: a systematic review, meta-analysis, and meta-regression. JAMA Psychiat.

[30] Neil AL, Christensen H (2009). Efficacy and effectiveness of school-based prevention and early intervention programs for anxiety. Clin Psychol Rev.

[31] Stockings EA, Degenhardt L, Dobbins T, Lee YY, Erskine HE, Whiteford HA, Patton G (2016). Preventing depression and anxiety in young people: a review of the joint efficacy of universal, selective and indicated prevention. Psychol Med.

[32] Teubert D, Pinquart M (2011). A meta-analytic review on the prevention of symptoms of anxiety in children and adolescents. J Anxiety Disord.

[33] Silverman WK, Hinshaw SP (2008). The second special issue on evidence-based psychosocial treatments for children and adolescents: a 10-year update. J Clin Child Adolesc Psychol.

[34] Higgins E, O’Sullivan S (2015). “What Works”: Systematic review of the “FRIENDS for Life” programme as a universal school-based intervention programme for the prevention of child and youth anxiety. Educ Psychol Pract.

[35] Matsumoto Y, Shimizu E (2016). The FRIENDS Cognitive Behavioral Program in Japanese schools: an examination of the treatment effects. School Psychol Int.

[36] Kato S, Shimizu E (2017). A pilot study on the effectiveness of a school-based cognitive-behavioral anxiety intervention for 8-and 9-year-old children: a controlled trial in Japan. Ment Health Prev.

[37] Urao Y, Yoshinaga N, Asano K, Ishikawa R, Tano A, Sato Y, Shimizu E (2016). Effectiveness of a cognitive behavioural therapy-based anxiety prevention programme for children: a preliminary quasi-experimental study in Japan. Child Adolesc Psychiatry Ment Health.

[38] Urao Y, Yoshida M, Koshiba T, Sato Y, Ishikawa SI, Shimizu E (2018). Effectiveness of a cognitive behavioural therapy-based anxiety prevention programme at an elementary school in Japan: a quasi-experimental study. Child Adoles Psychiatry Ment Health.

[39] Ishikawa SI, Sato H, Sasagawa S (2009). Anxiety disorder symptoms in Japanese children and adolescents. J Anxiety Disord.

[40] Spence SH (1997). Structure of anxiety symptoms among children: a confirmatory factor-analytic study. J Abnorm Psychol.

[41] Muris P, Schmidt H, Merckelbach H (2000). Correlations among two self-report questionnaires for measuring DSM-defined anxiety disorder symptoms in children: the screen for Child Anxiety Related Emotional Disorders and the Spence Children's Anxiety Scale. Person Individ Differ.

[42] Ministry of Education, Culture, Sports, Science and Technology. The survey result on Children in regular school class with possible developmental disorder who need special educational support. https://www.mext.go.jp/a_menu/shotou/tokubetu/material/1328729.htm. Accessed 15 Aug 2019.

[43] Van Steensel FJ, Bögels SM, Perrin S (2011). Anxiety disorders in children and adolescents with autistic spectrum disorders: a meta-analysis. Clin Child Fam Psychol Rev..

[44] Collins S, Woolfson LM, Durkin K (2014). Effects on coping skills and anxiety of a universal school-based mental health intervention delivered in Scottish primary schools. School Psychol Int.

